# A Biomimetic Porcine Urothelial Model for Assessing *Escherichia coli* Pathogenicity

**DOI:** 10.3390/microorganisms10040783

**Published:** 2022-04-07

**Authors:** Luka Predojević, Darja Keše, Darja Žgur Bertok, Taja Železnik Ramuta, Peter Veranič, Mateja Erdani Kreft, Marjanca Starčič Erjavec

**Affiliations:** 1 Biotechnical Faculty, University of Ljubljana, SI-1000 Ljubljana, Slovenia; lukapredojevic@yahoo.com (L.P.); darja.zgur@bf.uni-lj.si (D.Ž.B.); 2 Faculty of Medicine, Institute of Microbiology and Immunology, University of Ljubljana, SI-1000 Ljubljana, Slovenia; darja.kese@mf.uni-lj.si; 3 Faculty of Medicine, Institute of Cell Biology, University of Ljubljana, SI-1000 Ljubljana, Slovenia; taja.zeleznik@mf.uni-lj.si (T.Ž.R.); peter.veranic@mf.uni-lj.si (P.V.)

**Keywords:** urothelium, virulence-associated genes, infection, electron microscopy, *E. coli*, UPEC, correlations, pathogenicity groups, primary culture, infection assay

## Abstract

Urinary tract infections can be severe, sometimes fatal, diseases whose etiological pathogens are predominantly uropathogenic strains of *E. coli* (UPEC). To investigate the UPEC pathogenesis, several models have already been established with minor or major disadvantages. The aim was to develop a simple, fast, and inexpensive biomimetic in vitro model based on normal porcine urothelial (NPU) cells that are genetically and physiologically similar to human bladder urothelium and to perform basic studies of *E. coli* pathogenicity. Initially, the model was tested using a set of control *E. coli* strains and, subsequently, with human *E. coli* strains isolated either from patients with urinary infections or from the feces of healthy individuals. A drop in viability of NPU cells was used as a measure of the pathogenicity of the individual strain tested. To visualize the subcellular events, transmission and scanning electron microscopy was performed. The strains were tested for the presence of different virulence-associated genes, phylogroup, type of core lipid, O-serotype, and type of lipopolysaccharide and a statistical analysis of possible correlations between strains’ characteristics and the effect on the model was performed. Results showed that our model has the discriminatory power to distinguish pathogenic from non-pathogenic *E. coli* strains, and to identify new, potentially pathogenic strains.

## 1. Introduction

*Escherichia coli* (*E. coli*) is a Gram-negative, usually motile, facultative anaerobic [1], rod-shaped [2], non-sporulating bacterium [3]. *E. coli* resides in the lower part of the digestive system of animals with constant body temperature including humans [4]—more precisely, in the mucus layer of the colon [5]. It benefits the human host by producing vitamin B12 [6] and vitamin K [7]. Vitamin K is essential for blood clotting and other biological processes such as, for example, prevention of oxidative stress in neurons and rescue of mitochondrial dysfunction by facilitating ATP synthesis [8]. Finally, *E. coli* also protects the human gut from colonization by pathogenic bacteria [9,10]. *E. coli* is therefore considered to be a commensal microorganism with a mutualistic relationship with its host.

Most *E. coli* strains are considered harmless [11], but pathogenic *E. coli* strains that produce virulence factors such as adhesins, toxins, invasins, iron acquisition systems, and capsules can cause different types of infections [5]. Furthermore, bacterial cell structures stimulate cytokine production, relevant for the immune response, in host cells. Among pathogenic *E. coli* strains, two main groups are distinguished: intestinal pathogenic strains and extra intestinal pathogenic strains (ExPEC) [12]. Among ExPEC, the uropathogenic (UPEC) strains that can colonize and cause urinary tract infections (UTIs) in humans represent a serious global health problem [13,14]. UPEC strains are responsible for 75–95% of community-acquired UTIs [15]. UTIs are one of the most common types of infection in women. Anatomical factors can be responsible for infection of the female urethra by fecal microorganisms [16]. UTIs in women can be painful [17], often recurrent [16], and can lead to severe complications such as pyelonephritis, which can cause potentially irreversible damage of the kidneys and even death [18].

Many different models for assessing various aspects of uropathogenicity have already been established, each with advantages and limitations. The existing models for the investigation of UPEC pathogenicity encompass a diverse range of model organisms, including simple worms such as the nematode *Caenorhabditis elegans* and insects such as the wax moth (*Galleria mellonella*), but also higher organisms such as fish (*Danio rerio*), birds, mice, and pigs [19,20]. The above-mentioned models have their advantages, such as real-time observation of infection progress in vertebrates, as in transparent fish embryos (*D. rerio*) [20] or applicability of cultivation at optimal *E. coli* temperature (above 37 °C), as in the larvae of the insect *G. mellonella* model [21]. On the other hand, there are unavoidable limitations—low optimal growth temperature for fish embryos, absence of urinary bladder in birds, phylogenetic distance to humans (nematode and insect models), or the complicated, expensive, and slow process of growing and using large animals such as pigs [20]. In contrast, murine model systems are widely used in research focusing on urinary tract infections caused by UPEC since they can provide insight into many aspects of UPEC pathogenesis [22,23]. As mentioned, pigs can be used as a macro model system in vivo, although this is problematic in terms of maintenance. The big advantage of using pigs is their biological closeness to the human organism [24]. Moreover, the anatomical similarity enables the use of human medical equipment such as cystoscopes and urinary catheters to monitor the progression of infection in pigs [25].

A widely used alternative for studying the consequences and mechanisms of interactions between UPEC strains and host cells are cell cultures [20]. The greatest advantage of in vitro model systems is the possibility to perform the experiments in a highly controlled physical-chemical environment (temperature, pH, O_2_ and CO_2_ partial pressure, etc.) [26]. Further advantages are the possibility of genetic manipulation [27] and biochemical analysis [28]. Cell cultures are often grown in monolayers [29,30], but cells can also be stimulated to form multiple layers and reach partial or high differentiation states by cultivation in specific culture conditions, media, and scaffolds [31,32,33]. In vitro models for studying UPEC pathogenicity are usually prepared from normal primary cell cultures [33,34,35,36,37] or cancerous cell lines, originating from tissues such as bladder urothelium [31,38]. Human immortalized urothelial cell cultures have already been used as a model for studying the formation of intracellular clusters of bacteria among UPEC strains [39], as well as bacterial invasion, escape, and secondary infection [40]. Another interesting approach using cell cultures is also the bladder-chip model, which mimics the architecture of the human bladder and is constructed as a co-culture of two human cell lines—the human bladder carcinoma cell line HTB-9 and microvascular endothelial cells where both cell types are exposed to urine and cell growth medium [41].

A new alternative in vitro approach to understanding bacterial pathogenesis is also the human urothelial organoid model established using HBEP and HBLAK cells, which were not isolated from a normal urinary bladder but from bladder trigone biopsies from male patients undergoing surgery for benign prostatic hyperplasia. However, both cell types have an ability to form urothelium-like constructs with three distinctive layers and with uppermost layer of umbrella-like cells. Although some superficial cells expressed uroplakin 3 (UPIII), the ultrastructure of these cells was at the level of poorly differentiated urothelial cells with microvilli and some ropy ridges, and without uroplakin-positive urothelial plaques at the apical plasma membrane. The cells have no fusiform vesicles [42]. Since the urinary tract is exposed to a variety of possible infections and injures that may lead to organ damage or loss, the establishment of a biomimetic urothelial model with molecular, ultrastructural, and physiological characteristics of normal bladder urothelium for the in vitro study of uropathogenicity and corresponding cellular responses is necessary.

In our previous studies, we established the in vitro model of normal, non-cancerous, urothelial cells derived from porcine urinary bladder urothelium to study changes in cell proliferation, differentiation, and the formation or regeneration of urothelial permeability barriers after mechanical or chemical injuries [32,43]. The morphology, molecular composition, and ultrastructure of the porcine urothelial model were thoroughly characterized in our previous studies [32,43,44,45,46,47,48]. Briefly, normal porcine urothelial (NPU) cells in the model are connected with tight well-developed junctions and maintain high transepithelial resistance (a mean TEER of 4496 ± 291 Ωcm^2^, [32]). For comparison, the barrier between urine and blood has been shown to exhibit the highest recorded TEER of all epithelia with a value of up to 78,000 Ωcm^2^ [49]. Nevertheless, other measurements of different bladder urothelia demonstrated more than 10 times lower TEER values. The values were obtained from excised mucosa from different donor tissues and ranges between 1250 Ωcm^2^ (rat) to 5800 Ωcm^2^ (mouse) [50]. As far as we know, for porcine and human urothelium in vivo or ex situ there is no data for TEER in the literature, but the permeability values are known. Urothelium of the urinary bladder is consistently the least permeable epithelium among most organisms, and the permeability coefficients are for porcine urothelium 8.8 × 10^−5^ cm/s and human urothelium 7.7 × 10^−5^ cm/s [51,52] (in vitro data). Extensive electron microscopy analyses of this model revealed that apical plasma membrane of superficial urothelial cells is mainly shaped in rounded ridges and rarely in microvilli. In addition, the immunofluorescence labeling of cryo semi-thin sections with antibodies against UPIa, UPIb, UPII, and UPIIIa showed positive signals of all four UPs in superficial NPU cells. Importantly, all four UPs in the apical plasma membrane of NPU cells were additionally confirmed by the freeze-fracture replica immunolabeling (FRIL) technique. FRIL showed that anti-UPs antibodies recognized intramembrane UP-positive particles, which were organized into urothelial plaques ([44]). Altogether, these results show that endogenously expressed UPs not only target the apical plasma membrane of NPU cells in vitro, but also form urothelial plaques in these cells, which has not been shown for other urothelial in vitro models. This is also important because UPIa is a receptor for UPEC. Moreover, since our porcine urothelial model, on which this model system is based, has a high genetic and physiological similarity to the human urothelium, there is a high probability that the results obtained with this model are also valid for human medicine. It is important to note that it is difficult to obtain normal human bladder urothelium for ethical reasons. Therefore, in this study we have used porcine urothelium to develop an alternative model system for studying the uropathogenesis of human *E. coli* strains that is biomimetic and has a high-resolution capacity as it also express UPIa, which is a receptor for UPEC [53].

In the presented study, the biomimetic normal porcine urothelial in vitro model was initially used in infection experiments with well-defined and characterized *E. coli* strains. The NPU cell viability was determined and transmission (TEM) and scanning electron microscopy (SEM) were performed. Subsequently, the efficacy of the model system was tested also on a larger set of *E. coli* strains isolated either from patients with UTI or from fecal samples of healthy human individuals. Our goal was to evaluate if the porcine urothelial cells in vitro respond to infection with various human *E. coli* strains.

## 2. Materials and Methods

### 2.1. NPU Cell Cultures

For the construction of our in vitro biomimetic model, normal healthy urothelial cells derived from the normal porcine bladder urothelium were used. Porcine urinary bladders were obtained from a local slaughterhouse. The experiments were approved by the Veterinary Administration of the Slovenian Ministry of Agriculture and Forestry in compliance with the Animal Health Protection Act and the Instructions for Granting Permits for Animal Experimentation for Scientific Purposes. Normoplastic urothelial models of normal porcine urothelial cells (NPU cells) were established as previously described [32,43,46] and in compliance with the guidelines for replacement, reduction, and refinement (3Rs) in laboratory animal use. The porcine urinary bladder was cut into 5 cm long and 2 cm wide strips. The urothelium was gently scraped with a scalpel blade and filtered through a 40 μL Cell Strainer (BD Falcon, Bedford, MA, USA), and cells were seeded onto polystyrene tissue culture flasks (TPP, Trasadingen, Switzerland) at a density of 2 × 10^5^ cells/cm^2^. The NPU cells were grown in UroM medium, which consisted of equal parts of MCDB153 medium (Sigma-Aldrich, Taufkirchen, Germany) and advanced Dulbecco’s modified essential medium (Invitrogen, Molecular Probes, Leiden, The Netherlands), and was supplemented with 0.1 mM phosphoethanolamine (Sigma-Aldrich), 15 μg/mL adenine (Sigma-Aldrich), 0.5 μg/mL hydrocortisone (Sigma-Aldrich), 5 μg/mL insulin (Sigma-Aldrich), 4 mM glutamax (Gibco, ThermoFisher, Waltham, MA, USA), 100 μg/mL streptomycin, and 100 U/mL penicillin.

At 80–100% confluence, the NPU cells were harvested with TripLE™ Select (Gibco) and re-seeded. The NPU cells from the VI to X passage were seeded onto 12-well culture inserts with 0.4 μm porous membranes and 0.9 cm^2^ effective growth areas (BD Falcon) at a seeding density of 2 × 10^5^ cells/cm^2^. The NPU cells were maintained in UroM medium with 0.9 mM extracellular calcium concentration and 2.5% fetal bovine serum (FBS), i.e., UroM (−Ca^2+^ + FBS) until confluence. For the establishment of the hyperplastic model with partially differentiated UCs, cells remained in UroM (−Ca^2+^ + FBS). For the establishment of the normoplastic model with highly differentiated urothelium, NPU cells were transferred after 1 week into UroM medium with 2.5 mM calcium without serum, i.e., UroM (+Ca^2+^ − FBS), and cultured for 3 to 6 weeks at 37 °C in a humidified 5% CO_2_ atmosphere (CO_2_ incubator, Thermo Scientific, Heraeus^®^, Waltham, MA, USA).

### 2.2. Bacterial Strains

In initial assays, to set up the biomimetic porcine urothelial cell culture model, well-known, already thoroughly described, and fully sequenced *E. coli* strains were used: two uropathogenic (UPEC) strains J96 and 536 isolated from UTI patients, one natural human commensal strain SE15 isolated from faecal samples of a healthy human donor, and the laboratory strain MG1655 (Table 1).

In subsequent infection assays, performed to evaluate the model, 10 randomly selected *E. coli* strains from the collection of natural human fecal strains (FEC) isolated from the feces of healthy volunteer individuals at the Department of Biology, Biotechnical Faculty, Slovenia and 10 randomly selected strains from the collection of natural human *E. coli* strains isolated from the urine of UTI patients at the Institute of Microbiology and Immunology, Faculty of Medicine, University of Ljubljana, Slovenia were used (Table 1).

### 2.3. Infection Assay

Bacterial cells, kept at −80 °C, were inoculated into 5 mL of Luria broth (LB) medium (LLG Labware, Meckenheim, Germany) and grown overnight with aeration (180 rpm) at 37 °C. The next day, 100 μL of the bacterial overnight culture was transferred into 10 mL of LB medium and incubated for 3 h at 37 °C and 5% CO_2_ without aeration. Then, using nephelometer, the bacterial cultures were diluted in saline solution (0.9% NaCl) to 0.5 McFarland, centrifuged (10 min, 4000× *g*) and resuspended in UroM growth medium to the final concentration of 10^7^ bacterial cells/mL and used with the multiplicity of infection 10:1 in infection assays on the NPU cells grown on plastic 24-multiwell plates.

After a 3 h (in preliminary experiments also 1 and 15 h) incubation (37 °C, 5% CO_2_, without aeration) the supernatant from each well was removed and wells were washed 3× with 1 mL of serum-free UroM growth medium with calcium, then 0.5 mL of commercially available proteolytic TrypLE Select Enzyme (Fisher Scientific, Gibco, Vienna, Austria) and incubated for approx. 40–60 min at 37 °C, 5% CO_2_. Afterwards, 0.5 mL of UroM medium was added directly into each well, cells were gently stirred, transferred into 1.5 mL Eppendorf tubes, centrifuged for 5 min at 200× *g* and gently resuspended in 1 mL UroM medium. Fifty μL of prepared cell suspension was mixed with 10 μL of 0.4% trypan blue dye. Using a hemocytometer, the number of dead and live NPU cells was counted in four quadrants under the inverted light microscope. The viability of NPU cells (%) was determined by the formula Equation (1):

(1) $$\mathrm{Viability}\;\mathrm{of}\;\mathrm{NPU}\;\mathrm{cells}\;\left(\%\right)=\frac{\;nL_{infected\;cells}}{\;{\left(nL+nD\right)}_{negative\;control}\;}\;\times \;100$$

Equation (1). Establishment of the viability of NPU cells. *nL_infected cells_* = number of all live NPU cells, when infected with the tested bacterial strain, counted in four quadrants on the hemocytometer. (*nL* + *nD*)*_negative control_* = number of all live (*nL*) and dead (*nD*) NPU cells counted in four quadrants on hemocytometer in the negative control.

In each experiment, to test the model that was initially set up with the control strains (J96, 536, SE15, MG1655), all the control strains alongside the other *E. coli* strains were used. Details on the choice of strains for each experiment are presented in Supplementary Table S1.

To make all values of NPU cell viability comparable between independent experiments, all values were corrected according to the value of the negative control (NPU cells without bacteria) in each independent experiment.

### 2.4. Scanning and Transmission Electron Microscopy

For the evaluation of the bacterial strain’s ability to attach and invade NPU cells in urothelial models scanning (SEM) and transmission (TEM) electron microscopy was performed as described previously [32,43,44]. Briefly, the models were fixed in 4% paraformaldehyde (*w*/*v*) and 2.5% glutaraldehyde (*v*/*v*) in 0.1 M cacodylate buffer, pH 7.4 for 2 h 45 min at 4 °C. The fixation was followed by overnight rinsing in 0.1 M cacodylate buffer. For TEM, the samples were then postfixed in 1% (*w*/*v*) osmium tetroxide for 1 h at 4 °C, dehydrated in a graded series of ethanol, and embedded in Epon (Serva Electrophoresis, Heidelberg, Germany). Ultrathin sections were contrasted with uranyl acetate and lead citrate and examined with the transmission electron microscope Philips CM100 (Philips, Eindhoven, The Netherlands), operation voltage 80 kV, equipped with CCD camera (AMT, Danvers, MA, USA). For SEM, the urothelial models were after postfixation in 1% (*w/v*) osmium tetroxide for 1 h at 4 °C and dehydration in a graded series of ethanol, dried in hexamethyldisilazane (HMDS, Sigma-Aldrich) spattered with gold, and examined with a Vega 3 scanning electron microscope (Tescan, Brno, Czech Republic) at 20 kV.

### 2.5. PCR Profiling of Bacterial Strains

Phylogroups of the BJ, HS, and DL *E. coli* strains used in this study were determined using two methods, by the triplex PCR [57] and by the quadruplex PCR [58]. The presence of the number of virulence-associated genes was determined by specific primers for toxin genes (*cnf1*, *hlyA*, *usp*, *clbAQ*, *vat*), adhesin genes (*fimH*, *papGII*, *papGIII*, *afa/draBC*, *sfaDE*, *iha*, *yfcV*), iron acquisition genes (*fyuA*, *hbp*, *ireA*, *picU*, *iucD*, *iroN*), protectin genes (*kpsMTII*, *ompT*, *ompT-APEC*, *tcpC*, *traT*, *iss*, *neuB*), and invasin genes (*ibeA*) (Supplementary Table S2). The allele-specific PCR-based method [59] was also used to assign the *E. coli* strains into 12 different principal O-serotypes (O1, O2, O4, O6, O7, O12, O15, O16, O18, O25, O75, and O157). The prevalence of the five types of hetero-oligosaccharides (R1, R2, R3, R4, and K-12) in the bacterial lipopolysaccharide (LPS) was determined as described by [60].

### 2.6. LPS Profiling of Bacterial Strains

LPS extraction and SDS-PAGE of extracted LPS were essentially performed as described by Hitchcock and Brown 1983 [61], with the exception that a 3% stacking gel and a 10% separating gel were used and that the gel was run for 45–60 min at 120 V. The silver staining of the SDS-PAGE gels was performed as described by Tsai and Frasch 1982 [62], with the exception that only 5 min wash steps were performed and that staining was stopped with short incubation in a stop buffer (7% acetic acid in water).

### 2.7. Statistical Analysis

Statistical analysis of possible correlations between different types of data was carried out using software for performing the Fischer’s exact test [63], followed by Bonferroni correction. Statistical significance of similarities or differences was taken into account for all *p*-values below 0.05.

## 3. Results

### 3.1. Response of the Biomimetic Urothelial In Vitro Model to Infection with Control E. coli Strains

To set-up our in vitro model, we first performed infection assays using a set of control *E. coli* strains (J96, 536, SE15, and MG1655) and three different incubation times for infection (1, 3, and 15 h). The viability of NPU cells after infection with control strains was the main criterion for assessing the strain’s pathogenicity. In the assays with 1 h incubation of the model with bacteria there were no obvious differences in viability of NPU cells after the infection with each bacterial strain, especially having in mind the expected pathogenic nature of two UPEC strains J96 and 536. On the other side, experiments with 15 h incubation of the model with bacteria showed, that the number of living NPU cells had decreased too greatly, that again no meaningful difference was observed (Supplementary Table S3).

**Figure 1:**
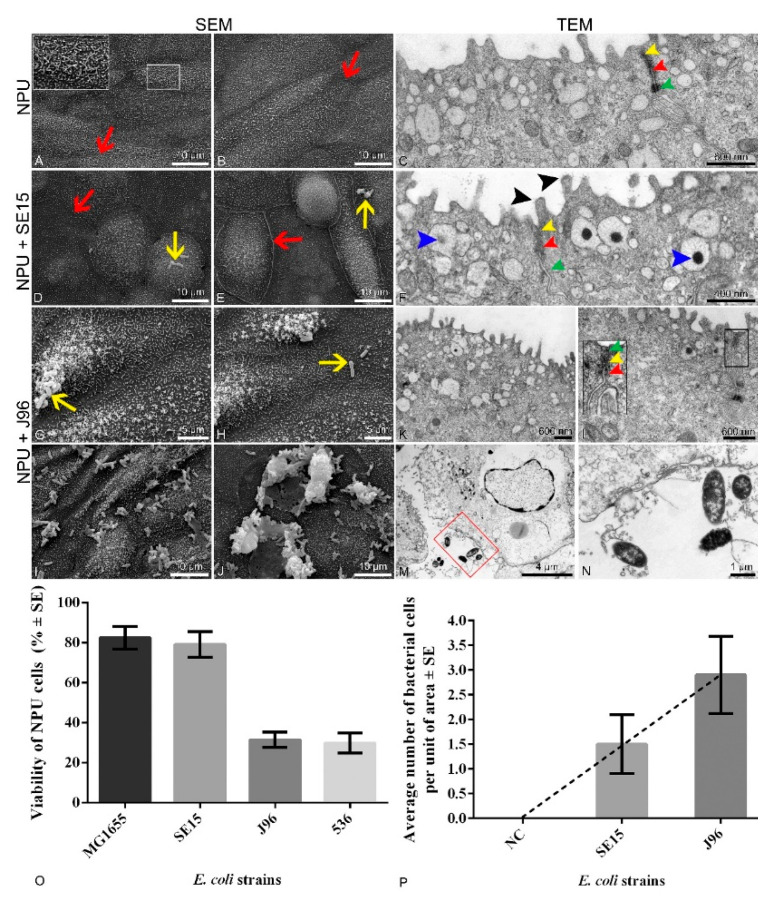
Apical plasma membrane surface topography of the normal porcine urothelial (NPU) cells of biomimetic urothelial in vitro model, captured by scanning electron microscope (SEM), and the micrographs of the cellular ultrastructure of the model captured using transmission electron microscope (TEM). In vitro model (**A**–**C**) incubated without bacteria (negative control). Borders of adjacent urothelial cells ((**A**,**B**), red arrows) are seen, as well as a network of intertwined surface microvilli ((**A**), white line inset). Tight junctions are normally developed ((**C**,**F**,**L**), yellow arrowheads) as well as adherent junctions ((**C**,**F**,**L**), red arrowheads) and desmosomes ((**C**,**F**,**L**), green arrowheads). Overview of the surface of the in vitro model infected with human commensal SE15 *E. coli* strain (**D**,**E**) shows the presence of rare bacterial cells per unit of the NPU cell apical surface (yellow arrows). There were no detected bacterial cells in the cytoplasm of NPU cells infected with SE15 (**F**). Glycocalyx can be seen on the surface of the microvilli ((**F**), black arrowheads), as well as dark aggregates discoidal or fusiform-shaped vesicles (DFVs) ((**F**), blue arrowheads). The SEM micrographs of the apical surface of the in vitro model infected with the human uropathogenic J96 *E. coli* strain show the presence of attached individual ((**G**,**H**), yellow arrows) or groups of bacteria (**I**,**J**) in a much higher number per unit of the apical area compared to the models infected with SE15. (**K**) In rare cases the morphology of urothelial cells incubated with J96 has been preserved. Cell junctions were preserved in only a few regions ((**L**), black line inset) and were disrupted in most cases, as is seen in (**M**). J96 disrupted the normal morphology of the urothelium, and J96 bacteria were present also in the extracellular matrix (**M**,**N**). Image (**N**) is an enlarged red square marked in figure (**M**). Insets framed with white or black are 200% enlarged, corresponding to smaller white or black insets. Two independent experiments were performed to examine the effect of different bacterial strains on ultrastructural characteristics of NPU cells. The representative micrographs are shown. (**O**) Percentage of viable NPU cells following infection with a set of control non-pathogenic (MG1655 and SE15) strains and pathogenic (J96 and 536) *E. coli* strains. Results were obtained from four biological samples in altogether at least 10 independent experiments. Error bars represent standard error of the mean (SE). (**P**) Number of individual bacterial cells attached to the apical cell surface of the in vitro model per unit of area. Unit of area was 77.6 × 77.6 µm. Bacterial cells on the SEM micrographs were counted in 50 different areas. Results were obtained from one biological sample in one independent experiment performed in triplicate. Error bars represent standard error of the mean (SE). Analysis of the number of individual bacterial cells of *E. coli* strains SE15 and J96 per unit of area in the in vitro model using the t-test did not show a statistically significant difference. Nevertheless, the average number of attached uropathogenic J96 bacterial cells is evidently higher, as shown by the trend line. Negative control (NC) represents data from the model grown without bacteria.

The results of the initial assays using a set of control strains: the uropathogenic strains J96 and 536 (pathogenic, control strains) and the laboratory MG1655 and human commensal SE15 strains (non-pathogenic, control strains) and the 3 h incubation period for infection are shown in Figure 1 and Supplementary Table S1. Both well-characterised human uropathogenic strains, J96 and 536, caused a drop in viability of the NPU cells, while the benign, non-pathogenic strains MG1655 and SE15, as expected, caused no drop in NPU cell viability. Based on the viability of NPU cells results showed clear distinction between pathogenic and non-pathogenic effect of *E. coli* strains.

Bacterial adhesion to NPU cells and possible ultrastructural changes of NPU cells due to 3 h bacterial infection were determined by both scanning and transmission electron microscopy.

Furthermore, the efficacy of the in vitro model was addressed by performing infection assays (3 h incubation period for infection) using a broader collection of *E. coli* strains, both isolated from the urine of UTI patients and fecal strains, in addition to the control strains mentioned above.

### 3.2. Ultrastructural Changes of the Biomimetic Urothelial Model

Images captured by SEM and TEM (Figure 1) revealed differences in the number of uropathogenic bacterial cells attached to the apical plasma membrane of superficial urothelial cells. Infection assays revealed that the human uropathogenic *E. coli* strain J96 was attached to the apical surface of the urothelial model in a much higher number of individual bacterial cells than human fecal strain SE15 (Figure 1, Supplementary Table S4). Moreover, SEM and TEM also revealed J96 intracellularly in endocytotic compartments. Furthermore, *E. coli* strain J96 caused pathological ultrastructural changes of the urothelial model, affecting the integrity of NPU cells, and thus the occurrence of J96 bacteria extracellularly, was due to cell lysis. Such ultrastructural changes affecting the integrity of NPU cells were not seen in in vitro models grown without bacteria and as such they were used as a negative control.

Analysis of the apical surface of the NPU cells using SEM revealed, a higher number of attached *E. coli* bacterial strain J96 per unit of the apical surface compared to the apical surface of NPU cells infected with fecal strain SE15. The results of the analysis are presented in Figure 1. To sum up, uropathogenic bacteria are attached and endocytosed into normal urothelial cells of our biomimetic model.

### 3.3. Response of the Biomimetic Urothelial In Vitro Model to Infection with a Set of 20 Randomly Selected Human E. coli Strains

**Figure 2:**
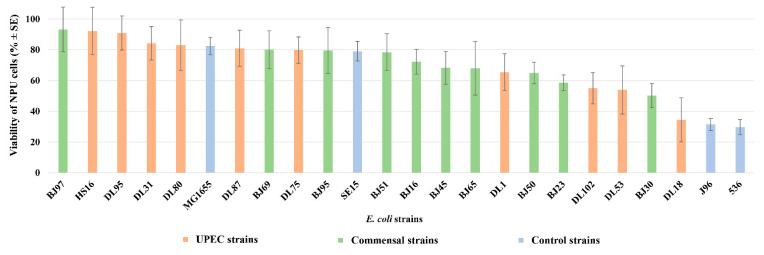
Viability of NPU cells infected with 20 different natural and a set of control pathogenic and non-pathogenic *E. coli* strains. Human *E. coli* strains isolated from the fecal samples of healthy individuals are designated as BJ, (green columns); human *E. coli* strains isolated from patients with urinary tract infection are designated as DL or HS (red columns); set of control pathogenic (J96, 536) and non-pathogenic (MG1655, SE15) *E. coli* strains (blue columns). Results were obtained on four biological samples in altogether from 5 to 22 independent experiments. Error bars represent standard error of the mean (SE).

To confirm the efficiency and the usability of the in vitro model to differentiate pathogenic and non-pathogenic human *E. coli* strains, a set of 20 randomly selected human *E. coli* strains was employed in infection assays. Ten strains were selected from a larger collection of human urinary clinical isolates from patients with UTIs, as well as 10 human fecal *E. coli* strains isolated from fecal samples of healthy individuals. As seen in Figure 2, the pathogenic effect of the two groups of strains on the viability of NPU cells was not clearly separated, as a mixed distribution of strains across the graph is evident.

### 3.4. Statistical Analysis of Correlation between Strain’s Pathogenicity Group and Virulence-Associated Genes, Bacterial LPS Type, O-serotype and Core-oligosaccharide Type

To perform the statistical analysis, the 24 tested *E. coli* strains (10 human *E. coli* strains isolated from patients with urinary tract infection, 10 *E. coli* strains isolated from fecal samples of healthy individuals, and a set of four control pathogenic and non-pathogenic strains) were, according the determined average viability of infected NPU cells, assigned to three different pathogenicity groups: (i) commensal group I—commensal, non-pathogenic strains (BJ97, HS16, DL95, DL31, DL80, MG1655, DL87, BJ69, DL75, BJ95, SE15, and BJ51) with NPU viability values ranging from 100% to 75%, (ii) low pathogenic group II—low pathogenic strains (BJ16, BJ45, BJ65, DL1, and BJ50) that provoked a drop in the viability of NPU cells ranging from 74% to 65%, and (iii) highly pathogenic group III—highly pathogenic *E. coli* strains (BJ23, DL102, DL53, BJ30, DL18, J96, and 536), that provoked a drop in viability of NPU cells ranging from 64% to 0%. The Fisher exact test was performed to reveal any statistical correlation between the assigned pathogenicity groups and the presence of virulence-associated genes and other strains’ characteristics (Supplementary Table S5). As seen from Table 2 and Supplementary Table S6, several virulence-associated genes revealed strong positive correlation with the most pathogenic *E. coli* strains (highly pathogenic group III). Positive correlation between highly pathogenic group III and the following virulence-associated genes, *cnf1*, *hlyA*, *clbAQ*, *papGIII*, *sfaDE*, and *tcpC*, was revealed. As seen from Table 2, statistically significant correlations were observed between highly pathogenic group III and *cnf1*, *hlyA*, *clbAQ*, *papGIII*, *sfaDE*, and *tcpC* virulence-associated genes. Furthermore, the performed statistical analysis revealed that there were no statistically significant correlations between any of the three pathogenicity groups of strains with neither phylogroup, bacterial LPS type, O-serotype, nor core-oligosaccharide type.

## 4. Discussion

Our goal was to establish a biomimetic in vitro model system for studies of UPEC pathogenesis based on normal, non-cancerous, urothelial cells derived from porcine urinary bladder. The NPU cell model was thoroughly characterized in our previous studies [32,43,44,45,46,48,64], which revealed that the model is physiologically similar to the normal human urothelium. In initial experiments, well-defined *E. coli* strains (UPEC J96, UPEC 536, commensal SE15 strain, and laboratory MG1655 strain) were employed. Comparison of NPU cell viability after 3 h infection with these strains showed that the used biomimetic model was able to differentiate between pathogenic and non-pathogenic strains. The average viability of NPU cells infected with two non-pathogenic strains, MG1655 and SE15, was 82.4% and 79.05%, respectively, which was much higher compared to the lower average viability of NPU cells infected with two well-known uropathogenic strains J96 and 536, 31.42% and 29.72%, respectively.

The effects of control strains, commensal (SE15) and uropathogenic (J96), on NPU cells were also demonstrated by SEM and TEM. SEM revealed that both bacterial strains, SE15 and J96, were able to attach to the apical membrane of the NPU cells, but strain J96 was attached in much higher numbers per unit area than SE15. TEM revealed that only the bacterial strain J96 was able to invade the NPU cells. In addition, the bacterial strain J96 induced the cytotoxic effects on NPU cells, which led to disruption of the integrity of the NPU cell plasma membrane and pathological ultrastructural changes, confirming its pathogenic effect using our biomimetic urothelial model.

To sum up, the used biomimetic NPU cell culture model was shown to successfully distinguish UPEC strains from non-pathogenic commensal and laboratory strains. Therefore, the model system was subsequently used to analyze a randomly selected array of 10 *E. coli* strains isolated from the urine of UTI patients (DL/HS collection) and 10 *E. coli* strains isolated from feces of healthy individuals (BJ collection).

The 20 natural *E. coli* strains exhibited different effects on NPU cells. Following infection, NPU cell viability ranged from 93.21% to 29.72%. Hence, the model system was again able to distinguish pathogenic and non-pathogenic strains. Not unexpectedly, the drop in viability provoked by some of the clinical urinary strains (HS16, DL95, DL31, DL80, DL87, and DL75) was comparable to that of non-pathogenic strains. It is known that strains with low virulence potential can also be isolated from the urine of UTI patients [65], especially if the host is immunocompromised [66] or has renal abnormalities [67,68]. On the other hand, some BJ strains, for example (BJ30, possessing: *cnf1*, *hlyA*, *usp*, *clbAQ*, *vat*, *fimH*, *papGIII*, *sfaDE*, *yfcV*, *fyuA*, *iroN*, *kpsMTII*, *ompT*, *tcpC*, and *neuB*) from the collection of fecal *E. coli* strains, caused a drop in NPU cell viability comparable to pathogenic strains. Indeed, ExPEC strains are found among the normal gut microbiota of healthy individuals [69].

Some pathogenic strains also have the ability to evade the immune system [70]. Mechanisms range from simple electrostatic repulsion that enable bacterial cells to evade the host’s antimicrobial peptides and changes in structures as PAMPs or LPS, to more complex ones, such as targeting a host’s intracellular signal transduction pathways [71].

In general, we demonstrated that strains with a higher total number of virulence-associated genes caused a greater drop in the viability of NPU cells. Statistical analysis of the characteristics of all 24 strains using the Fisher exact test revealed statistically significant correlations between the highly pathogenic group III and the following virulence-associated genes: *cnf1*, *hlyA*, *clbAQ*, *papGIII*, *sfaDE*, and *tcpC*. These correlations were in concordance with the high pathogenicity nature of strains of this group since the aforementioned virulence-associated genes code for toxins, adhesins, and protectins. These results are also in accordance with previous studies of UPEC pathogenesis using various model systems [72,73], as well as studies of the association of virulence factor genes and UTIs (cystitis, pyelonephritis) [74,75]. Very interesting are the fecal *E. coli* strains isolated from healthy individuals (BJ50, BJ65, and BJ45) that are lacking known virulence-associated genes, but they were nevertheless able to cause decreased NPU cell viability. Such *E. coli* strains might possess novel, not yet discovered, virulence-associated genes. Further research (complete genome sequencing, combined with insertion mutagenesis of potential new virulence-associated genes) focused on such strains may lead to new, more profound understanding of the complexity of the UPEC uropathogensis.

## 5. Conclusions

To conclude, the described biomimetic urothelial in vitro model provides a simple, fast, and financially sustainable assessment of the pathogenicity of *E. coli* strains. Due to genetic, anatomical, and physiological similarities between pigs and humans, the results obtained using this model can be extrapolated to humans. The here-established model enables both pre-clinical testing of *E. coli* strain pathogenicity, as well as basic studies of *E. coli* pathogenicity mechanisms. Furthermore, testing of potential new antimicrobial agents can also be performed using this model. In addition, as UPEC are the predominant, but not the only causative agent of UTIs, the here-presented model could be employed for investigation of other pathogens/opportunists provoking UTIs. Results obtained using this model may hence be of multiple significances—clinical, commercial, and scientific.

## 6. Patents

L.P., D.K., M.E.K. and M.S.E., together with Marina V. Kuznetsova, are co-authors on the patent application with the title BIOMIMETIC in vitro MODEL OF PORCINE UROTHELIUM FOR ASSESSING THE PATHOGENICITY OF AVIAN PATHOGENIC STRAINS OF *Escherichia coli* FOR HUMANS (P-201900249) at the Slovenian patent office and at the Eurasian patent office.

## Figures and Tables

**Table 1:** Bacterial *E. coli* strains used in the study.

Designation of the Strain	Relevant Information on Strains’ Characteristic	Source/Reference
J96	Uropathogenic strain, used as a control strain	Eva Moreno
536	Uropathogenic strain, used as a control strain	Eva Moreno
SE15	Commensal strain, used as a control strain	Eric Oswald
MG1655	Laboratory strain, used as a control strain	Christophe Beloin
DL1	Isolated from urine of a UTI patient	[54]
DL18	Isolated from urine of a UTI patient	[54]
DL31	Isolated from urine of a UTI patient	[54]
DL53	Isolated from urine of a UTI patient	[54]
DL75	Isolated from urine of a UTI patient	[54]
DL80	Isolated from urine of a UTI patient	[54]
DL87	Isolated from urine of a UTI patient	[54]
DL95	Isolated from urine of a UTI patient	[54]
DL102	Isolated from urine of a UTI patient	[54]
HS16	Isolated from urine and blood of a UTI patient	[55]
BJ16	Fecal strain from a healthy volunteer	[56]
BJ23	Fecal strain from a healthy volunteer	[56]
BJ30	Fecal strain from a healthy volunteer	[56]
BJ45	Fecal strain from a healthy volunteer	[56]
BJ50	Fecal strain from a healthy volunteer	[56]
BJ51	Fecal strain from a healthy volunteer	[56]
BJ65	Fecal strain from a healthy volunteer	[56]
BJ69	Fecal strain from a healthy volunteer	[56]
BJ95	Fecal strain from a healthy volunteer	[56]
BJ97	Fecal strain from a healthy volunteer	[56]

**Table 2:** Distribution of virulence-associated genes among *E. coli* strain pathogenicity groups. Statistical analysis was performed on a set of 24 strains (10 human *E. coli* strains isolated from patients with urinary tract infection, 10 *E. coli* strains isolated from fecal samples of healthy individuals, and a set of 4 control pathogenic and non-pathogenic strains).

		Pathogenicity Group					
		Commensal Group I ^a^		Low Pathogenic Group II		Highly Pathogenic Group III	
		[no. (%)]		[no. (%)]		[no. (%)]	
Virulence-associated gene [no. (%)]		Group I [12 (50)]	Non-Group I ^b^ [12 (50)]	Group II [5 (21)]	Non-Group II ^c^ [19 (79)]	Group III [7 (29)]	Non-Group III ^d^ [17 (71)]
Toxins							
*cnf1*	[7 (29)]	1 (8)	6 (50)	1 (20)	6 (32)	5 (71) *	2 (12)
*hlyA*	[9 (38)]	2 (17)	7 (58)	1 (20)	8 (42)	6 (86) *	3 (18)
*usp*	[18 (75)]	9 (75)	9 (75)	2 (40)	16 (84)	7 (100)	11 (65)
*clbAQ*	[12 (50)]	4 (33)	8 (67)	1 (20)	11 (58)	7 (100) *	5 (29)
*vat*	[14 (58)]	6 (50)	8 (67)	1 (20)	13 (68)	7 (100)	7 (41)
Adhesins							
*fimH*	[23 (96)]	12 (100)	11 (92)	4 (80)	19 (100)	7 (100)	16 (94)
*papGII*	[5 (21)]	4 (33)	1 (8)	0 (0)	5 (26)	1 (14)	4 (24)
*papGIII*	[7 (29)]	1 (8)	6 (50)	1 (20)	6 (32)	5 (71) *	2 (12)
*afa/draBC*	[0 (0)]	0 (0)	0 (0)	0 (0)	0 (0)	0 (0)	0 (0)
*sfaDE*	[11 (46)]	3 (25)	8 (67)	1 (20)	10 (53)	7 (100) **	4 (24)
*iha*	[4 (17)]	2 (17)	2 (17)	0 (0)	4 (21)	2 (29)	2 (12)
*yfcV*	[17 (71)]	9 (75)	8 (67)	1 (20)	16 (84)	7 (100)	10 (59)
Iron acquisition systems							
*fyuA*	[17 (71)]	9 (75)	8 (67)	1 (20)	16 (84)	7 (100)	10 (59)
*hbp*	[2 (8)]	1 (8)	1 (8)	0 (0)	2 (11)	1 (14)	1 (6)
*ireA*	[6 (25)]	4 (33)	2 (17)	0 (0)	6 (32)	2 (29)	4 (24)
*picU*	[3 (13)]	2 (17)	1 (8)	0 (0)	3 (16)	1 (14)	2 (12)
*iucD*	[9 (38)]	7 (58)	2 (17)	0 (0)	9 (47)	2 (29)	7 (41)
*iroN*	[16 (67)]	8 (67)	8 (67)	1 (20)	15 (79)	7 (100)	9 (53)
Protectins							
*kpsMTII*	[14 (58)]	8 (67)	6 (50)	1 (20)	13 (68)	5 (71)	9 (53)
*ompT*	[19 (79)]	11 (92)	8 (67)	1 (20)	18 (95)	7 (100)	12 (71)
*ompT-APEC*	[7 (29)]	6 (50)	1 (8)	0 (0)	7 (37)	1 (14)	6 (35)
*tcpC*	[9 (38)]	2 (17)	7 (58)	1 (20)	8 (42)	6 (86) *	3 (18)
*traT*	[11 (46)]	7 (58)	4 (33)	1 (20)	10 (53)	3 (43)	8 (47)
*iss*	[5 (21)]	4 (33)	1 (8)	0 (0)	5 (26)	1 (14)	4 (24)
*neuB*	[8 (33)]	5 (42)	3 (25)	0 (0)	8 (42)	3 (43)	5 (29)
Invasins							
*ibeA*	[3 (13)]	1 (8)	2 (17)	0 (0)	3 (16)	2 (29)	1 (6)

^a^ To be able to perform statistical analysis, three pathogenic groups were formed: (i) commensal group I—commensal, non-pathogenic strains (BJ97, HS16, DL95, DL31, DL80, MG1655, DL87, BJ69, DL75, BJ95, SE15, and BJ51) with NPU viability values ranging from 100% to 75%, (ii) low pathogenic group II—low pathogenic strains (BJ16, BJ45, BJ65, DL1, and BJ50) that provoked a drop in the viability of NPU cells ranging from 74% to 65%, and (iii) highly pathogenic group III—highly pathogenic *E. coli* strains (BJ23, DL102, DL53, BJ30, DL18, J96, and 536) that provoked a drop in viability of NPU cells ranging from 64% to 0%. ^b^ Strains belonging either to low pathogenic group II or highly pathogenic group III, ^c^ strains belonging to either commensal group I or highly pathogenic group III, and ^d^ strains belonging to either commensal group I or low pathogenic group II. Statistically significant *p*-values after Bonferroni correction for positive correlations are marked with asterisks: * for *p*-values < 0.05, ** for *p*-values < 0.01.

## Data Availability

The authors confirm that the data supporting the findings of this study are available within the article and its Supplementary Materials. Data are freely available, under a license allowing re-use by any third party for any lawful purpose.

## Supplementary Materials

The following supporting information can be downloaded at https://www.mdpi.com/article/10.3390/microorganisms10040783/s1: Table S1: Details on the choice of strains for each experiment and relative viability values of urothelial cells obtained in different independent experiments; Table S2: PCR primers and programs; Table S3: Viability of NPU cells after 1 or 15 h incubation with control *E. coli* strains, references [76,77,78,79,80,81,82,83,84,85,86,87,88,89,90,91] are cited in the supplementary Table S4: Statistical analysis of the number of individual bacterial cells of strains SE15 and J96 attached to the surface of the in vitro model visualised using scanning electron microscopy; Table S5: Presence of virulence-associated genes (denoted with a dot), characteristics of LPS and phylogenetic group of the studied *E. coli* strains; Table S6A: P (two-tail) values obtained with automated Fisher exact test computer software (presented with four decimals); and Table S6B: Data obtained after Bonferroni correction applied to the data in the Supplementary Table S6A (presented with four decimals).
