# Supplementary material for: A Biomimetic Porcine Urothelial Model for Assessing Escherichia coli Pathogenicity
Source: Microorganisms. 2022 Apr 7;10(4):783. doi: 10.3390/microorganisms10040783 (PMC9029248; doi:10.3390/microorganisms10040783)
Supplement: Supplementary file 1 [file microorganisms-10-00783-s001.zip › microorganisms-1638435- supplementary correct.pdf]

A Biomimetic Porcine Urothelial Model for Assessing *Escherichia coli* Pathogenicity –

SUPPLEMENTAL MATERIAL

Luka Predojević[1], Darja Keše[2], Darja Žgur Bertok[1], Taja Železnik Ramuta[3], Peter Veranič[3], Mateja Erdani Kreft[3,*], Marjanca Starčič Erjavec[1,*]

Supplementary Table S1. Details on the choice of strains for each experiment and relative viability values of urothelial cells obtained in different independent experiments.

Supplementary Table S2. PCR primers and programs.

Supplementary Table S3. Viability of NPU cells after 1 or 15 h incubation with control *E. coli* strains.

Supplementary Table S4. Statistical analysis of the number of individual bacterial cells of strains SE15 and J96 attached to the surface of the *in vitro* model visualised using scanning electron microscopy.

Supplementary Table S5. Presence of virulence-associated genes (denoted with a dot), characteristics of LPS and phylogenetic group of the studied *E. coli* strains.

Supplementary Table S6A. P (two-tail) values obtained with automated Fischer's exact test computer software (presented with four decimals).

Supplementary Table S6B. Data obtained after Bonferroni correction applied to the data in the Supplementary Table S6A (presented with four decimals).

Supplementary Table S1. Details on the choice of strains for each experiment and relative viability values of urothelial cells obtained in different independent experiments.

		Independent experiment designation																												
Set of *E. coli* strains	Strain designation	LP10	LP11	LP13	LP14	LP15	LP16	LP17	LP18	LP19	LP20	LP22	LP23	LP24	LP25	LP26	LP27	LP28	LP29	LP30	LP31	LP33	LP34	Technical repeats	Number of independent experiments	Number of biological samples	Viability - average	Numerus	Standard deviation	Standard error
Control strains	J96	58.13	10.61	34.05	16.69	32.51	26.13	38.50	16.59	54.29	29.49	15.89	8.66	22.81	24.00	39.19	42.65	19.13	29.02	78.95	60.53	16.28	17.20	45	22	4	31.42	22	18.21	3.88
	536	35.08	14.00					41.50		71.43	14.74		18.56		18.00		28.44			34.65	24.21		26.29	31	11	4	29.72	11	16.46	4.96
	SE15		88.29					37.89		95.00	94.66		92.37		82.00	74.66				50.44	96.32	78.88		33	10	4	79.05	10	20.00	6.32
	MG1655	105.79	61.63	58.19	50.41	108.74	113.55	49.32	88.71	98.93	53.85	42.38	100.21	62.28	96.00	47.97	105.21	53.91	83.94	129.82	97.89	105.34	98.77	45	22	4	82.40	22	26.53	5.66
Fecal strains	BJ16	101.10			51.06			60.35		86.90	94.02	48.34						63.77						23	7	4	72.22	7	21.43	8.10
	BJ23				52.81	53.06		76.19				47.68						63.19						13	5	4	58.59	5	11.33	5.07
	BJ30			61.69		68.31			31.34		67.31	17.88						37.68			67.37			23	7	4	50.22	7	20.83	7.87
	BJ45				44.95	82.21		102.26				50.33						61.01						13	5	4	68.15	5	23.81	10.65
	BJ50				45.39	88.49		63.56				59.60						67.54						13	5	4	64.92	5	15.60	6.98
	BJ51			85.92			94.19	93.23				41.72						42.61		112.94				17	6	4	78.44	6	29.48	12.03
	BJ65				59.14		130.75	70.18				23.84						55.94						13	5	4	67.97	5	39.10	17.49
	BJ69			72.70			127.96		70.97			70.20						58.55						13	5	4	80.08	5	27.34	12.23
	BJ95			65.23			127.53	99.85				33.11						47.68		103.73				17	6	4	79.52	6	36.54	14.92
	BJ97				58.92		132.04	116.09				80.13						48.12			123.95			17	6	4	93.21	6	35.66	14.56
Uropathogenic strains	DL1			62.36		37.07			50.54					43.42					112.95		86.58			17	6	4	65.49	6	29.07	11.87
	DL18				10.04	19.13			29.65					23.25					90.16					13	5	4	34.44	5	31.95	14.29
	DL31			95.11		80.27			60.68					64.91					120.21					13	5	4	84.24	5	24.27	10.85
	DL53			112.36		23.47			23.04					20.18					83.94		60.26			17	6	4	53.87	6	38.41	15.68
	DL75			66.95		60.24			71.89					76.32					119.17	83.99				17	6	4	79.76	6	20.93	8.54
	DL80				32.73		127.10		67.59					78.07					109.33					13	5	4	82.96	5	36.83	16.47
	DL87				49.32		116.34		84.18					62.28					92.75					13	5	4	80.97	5	26.23	11.73
	DL95			84.20			125.16		71.43					77.19					63.21	124.34				17	6	4	90.92	6	27.10	11.06
	DL102				56.52		65.38		32.41					33.77					87.05					13	5	4	55.03	5	22.90	10.24
	HS16				46.70		130.75		77.88					83.33					122.28					13	5	4	92.19	5	34.44	15.40
	Animal/Passage	$P4U4_{a'}$	$P4U13_{a'}$	$P4U6_a1$	$P4U6_b$	$P4U9_a$	$P4U10_a$	$P2U4_c$	$P2U6_c$	$P2U9_c$	P3U9	P6U8	P6U11	P6U11	P3EU9	P3EU15	P3EU15	P3EU6	P3EU6	P3EU7	P3EU7	P6U8	P6U8							

Supplementary Table S2. PCR primers and programs.

Virulence-associated gene	Forward and reverse primer sequence (5'–>3')	PCR program		PCR product (bp)	Reference for the primer sequence
cnf1	CNF1-1 CTGACTTGCCGTGGTTTAGTCGG	94 °C - 4 min	1×	1295	Kuhar et al., 1998
		94 °C - 1 min 30 s<br>59 °C - 1 min 30 s<br>72 °C - 2 min	30×		
	CNF1-2 TACACTATTGACATGCTGCCCGGA	72 °C - 5 min	1×		
hlyA	hlyA.1 AACAAGGATAAGCACTGTTCTGGCT	95 °C - 2 min 30 s	1×	1177	Yamamoto et al., 1995
		94 °C - 30 s<br>64 °C - 30 s<br>72 °C - 1 min 30 s	30×		
	hlyA.2 ACCATATAAGCGGTCATTCCCGTCA	72 °C - 7 min	1×		
usp	USPdeg 1 ATGCTACTGTTTCCGGGTAGTSTGT	95 °C - 5 min	1×	1017	Vadnov et al., 2017
		95 °C - 30 s<br>56 °C - 30 s<br>72 °C - 1 min 30 s	30×		
	USPdeg 2 CATCRTGTAGTCKGGGSGTAACAAT	72 °C - 7 min	1×		
clbA	IHAPJPN42 CAGATACACAGATACCATTCA	94 °C - 4 min 30 s	1×	1002	Johnson et al., 2008
		94 °C - 30 s<br>57 °C - 30 s<br>72 °C - 1 min	30×		
	IHAPJPN46 CTAGATTATCCGTGGCGATTC	72 °C - 10 min	1×		
clbQ	IHAPJPN55 TTATCCTGTTAGCTTTCGTTC	94 °C - 4 min 30 s	1×	821	Johnson et al., 2008
		94 °C - 30 s<br>57 °C - 30 s<br>72 °C - 1 min	30×		
	IHAPJPN56 CTTGTATAGTTACACAACTATTTC	72 °C - 10 min	1×		

vat	vat-sp F<br>TCAGGACACGTTCAGGCATTCAGT<br><br>vat-sp R<br>GGCCAGAACATTTGCTCCCTTGTT	95 °C - 15 min 1×<br>94 °C - 30 s<br>63 °C - 1 min 30 s 30×<br>72 °C - 1 min 30 s<br>72 °C - 10 min 1×	1100	Vigil et al., 2011
fimH	FimH1<br>CAGCGATGATTTCCAGTTTGTGTG<br><br>FimH2<br>TGCGTACCAGCATTAGCAATGTCC	94 °C - 2 min 30 s 1×<br>94 °C - 30 s<br>64 °C - 30 s 30×<br>72 °C - 30 s<br>72 °C - 7 min 1×	461	Starčič Erjavec et al., 2011
papGII	papG_II f<br>GGGATGAGCGGGCCTTTGAT<br><br>papG_II r<br>CGGGCCCCCAAGTAACTCG	95 °C - 2 min 30 s 1×<br>95 °C - 30 s<br>55 °C - 1 min 30×<br>72 °C - 30 s<br>72 °C - 7 min 1×	190	Johnson and Brown, 1996
papGIII	papG_III f<br>GGCCTGCAATGGATTTACCTGG<br><br>papG_III r<br>CCACCAAATGACCATGCCAGAC	94 °C - 2 min 30 s 1×<br>94 °C - 30 s<br>63 °C - 30 s 25×<br>72 °C - 3 min<br>72 °C -10 min 1×	258	Johnson and Brown, 1996
afa/draBC	afa/draBC-f<br>GGCAGAGGGCCGGCAACAGGC<br><br>afa/draBC-r<br>CCCGTAACGCGCCAGCATCTC	94 °C - 4 min 1×<br>94 °C - 30 s<br>63 °C - 30 s 25×<br>72 °C - 3 min<br>72 °C - 10 min 1×	592	Johnson and Stell, 2000
sfaDE	SFA-1<br>CTCCGGAGAACTGGGTGCATCTTAC<br><br>SFA-2<br>CGGAGGAGTAATTACAAACCTGGCA	94 °C - 3 min 1×<br>94 °C - 2 min<br>65 °C - 1 min 25×<br>72 °C - 2 min<br>72 °C - 10 min 1×	408	Le Bouguenec et al., 1992

iha	iha f<br>CTGGCGGAGGCTCTGAGATCA<br><br>iha r<br>TCCTTAAGCTCCCGCGGCTGA	94 °C - 4 min 1×<br>94 °C - 30 s<br>58 °C - 30 s 30×<br>72 °C - 1 min<br>72 °C - 8 min 1×	827	Johnson et al., 2000
yfcV	yfcV F<br>ACATGGAGACCACGTTCACC<br><br>yfcV R<br>GTAATCTGGAATGTGGTCAGG	95 °C - 15 min 1×<br>94 °C - 30 s<br>63 °C - 1 min 30 s 30×<br>72 °C - 1 min 30 s<br>72 °C - 10 min 1×	292	Spurbeck et al., 2011
fyuA	fyuA 1[a]<br>TGATTAACCCCGCGACGGGAA<br><br>fyuA 2[b]<br>CGCAGTAGGCACGATGTTGTA	94 °C - 2 min 30 s 1×<br>94 °C - 30 s<br>63 °C - 30 s 25×<br>72 °C - 3 min<br>72 °C - 10min 1×	785	[a]Johnson et al., 2000; [b]Schubert et al., 1998
hbp	Hbp f<br>GGTGAAGGTACGCTGACGGT<br><br>Hbp r<br>GCGTGACGCTGGAGTTATCT	94 °C - 4 min 30 s 1×<br>94 °C - 30 s<br>65 °C - 1 min 35×<br>72 °C - 1 min<br>72 °C - 10 min 1×	925	Starčič Erjavec et al., 2009
ireA	ireA f<br>TGGTCTTCAGCTATATGG<br><br>ireA r<br>ATCTATGATTGTGTTGGT	94 °C - 2 min 30 s 1×<br>94 °C - 30 s<br>55 °C - 1 min 25×<br>72 °C - 30 s<br>72 °C - 10 min 1×	421	Russo et al., 2001
picU	picU f<br>TCAGGCCGGTAAGAACAGCAAAAT<br><br>picU r<br>ACGGTAAGAGTGTGGATGGCGGAGTC	94 °C - 2 min 1×<br>94 °C - 15 s<br>70 °C - 30 s 30×<br>72 °C - 45 s<br>72 °C - 10 min 1×	372	Parham et al., 2005

iucD	Aer1<br>TACCGGATTGTCATATGCAGACCGT<br>Aer2<br>AATATCTTCCTCCAGTCCGGAGAAG	94 °C - 4 min 30 s 1×<br>94 °C - 30 s<br>62 °C - 30 s 35×<br>72 °C - 50 s<br>72 °C - 10 min 1×	602	Yamamoto et al., 1995
iroN	iroN f<br>AAGTCAAAGCAGGGGTTGCCCG<br>iroN r<br>GACGCCGACATTAAGACGCAG	94 °C - 2 min 30 s 1×<br>94 °C - 30 s<br>68 °C - 30 s 25×<br>72 °C - 2 min<br>72 °C - 10 min 1×	668	Johnson et al., 2000
kpsMTII	kpsMT_II f<br>GCGCATTTGCTGATACTGTTG<br>kpsMT_II r<br>CATCCAGACGATAAGCATGAGCA	94 °C - 4 min 30 s 1×<br>94 °C - 30 s<br>60 °C - 1 min 25×<br>72 °C - 30 s<br>72 °C - 10 min 1×	270	Johnson and Stell., 2000
ompT	ompT f<br>ATCTAGCCGAAGAAGGAGGC<br>ompT r<br>CCCGGGTCATAGTGTTCATC	95 °C - 4 min 1×<br>94 °C - 30 s<br>57 °C - 1 min 25×<br>72 °C - 1 min<br>72 °C - 10 min 1×	559	Foxman et al., 1995
ompT-APEC	ompT-APEC f<br>CAGAGTATCTGTCGGTGCCTCA<br>ompT-APEC r<br>TACGGTTCCATGTTCCTTCGAC	94 °C - 4 min 30 s 1×<br>94 °C - 30 s<br>60 °C - 1 min 25×<br>72 °C - 1 min<br>72 °C - 10 min 1×	581	Vadnov et al., 2017
tcpC	tcpC-for<br>GGCAACAATATGTATAATATCCT<br>tcpC-rev<br>GCCCAGTCTATTTCTGCTAAAGA	94 °C - 4 min 30 s 1×<br>94 °C - 30 s<br>60 °C - 30s 25×<br>72 °C - 1 min<br>72 °C - 10 min 1×	386	Starčič Erjavec et al., 2010

traT	traT1<br>GGTGTGGTGCGATGAGCACAG<br>traT2<br>CACGGTTCAGCCATCCCTGAG	94 °C - 4 min 30 s 1×<br>94 °C - 30 s<br>71 °C - 30 s 30×<br>72 °C - 30 s<br>72 °C - 7 min 1×	288	Johnson end Stell, 2000
iss	Iss1<br>ACGATACTCCGTAGCCAGAGAT<br>Iss2<br>ATGAACAGTGCAGATGAGCTCC	95 °C - 4 min 30 s 1×<br>94 °C - 30 s<br>64 °C -30 s 30×<br>72 °C - 1 min<br>72 °C - 7 min 1×	793	Starčič Erjavec et al., 2011
neuB	neuB-F<br>CTACCCCTTTTGACGAAGAC<br>neuB-R<br>ACACACCTGACCCCAATAC	94 °C - 4 min 30 s 1×<br>94 °C - 30 s<br>64 °C - 30 s 30×<br>72 °C - 1 min<br>72 °C - 7 min 1×	493	Nowrouzian et al., 2001
ibeA	Ibe10_f<br>AGGCAGGTGTGCGCCGCGTAC<br>IbeA_r<br>TGGTGCTCCGGCAAACCATGC	94 °C - 2 min 30 s 1×<br>94 °C - 30 s<br>63 °C - 30 s 25×<br>72 °C - 3 min<br>72 °C - 10 min 1×	170	Johnson and Stell, 2000

Supplementary Table S3. Viability of NPU cells after 1 or 15 h incubation with control *E. coli* strains.

**1 h** incubation of the model with bacteria		**15 h** incubation of the model with bacteria	
E. coli strain	Viability of NPU cells (%)	*E. coli* strain	Viability of NPU cells (%)
J96	88.10	J96	15.76
536	133.56	536	13.93
SE15	121.80	SE15	22.82
MG1655	119.24	MG1655	7.66

Supplementary Table S4. Statistical analysis of the number of individual bacterial cells of strains SE15 and J96 attached to the surface of the *in vitro* model visualised using scanning electron microscopy.

**NC**		**SE15**		**J96**	
Image	Number of individual bacterial cells	Image	Number of individual bacterial cells	Image	Number of individual bacterial cells
1	0	1	0	1	3
2	0	2	0	2	0
3	0	3	0	3	0
4	0	4	0	4	0
5	0	5	0	5	2
6	0	6	0	6	2
7	0	7	0	7	1
8	0	8	0	8	32
9	0	9	0	9	3
10	0	10	0	10	2
11	0	11	0	11	13
12	0	12	1	12	17
13	0	13	4	13	4
14	0	14	8	14	7
15	0	15	28	15	1
16	0	16	2	16	0
17	0	17	6	17	1
18	0	18	0	18	7
19	0	19	6	19	2
20	0	20	2	20	0
21	0	21	0	21	6
22	0	22	0	22	5
23	0	23	1	23	9
24	0	24	0	24	6
25	0	25	1	25	6
26	0	26	3	26	1
27	0	27	2	27	0
28	0	28	3	28	0
29	0	29	2	29	0
30	0	30	1	30	0
31	0	31	2	31	0
32	0	32	1	32	1
33	0	33	0	33	0
34	0	34	0	34	1
35	0	35	0	35	0
36	0	36	2	36	0
37	0	37	0	37	0
38	0	38	0	38	0

39	0
40	0
41	0
42	0
43	0
44	0
45	0
46	0
47	0
48	0
49	0
50	0
Average	**0**
Count	50
SD	0
SE	0

39	0
40	0
41	0
42	0
43	0
44	0
45	0
46	0
47	0
48	0
49	0
50	0
Average	**1.5**
Count	50
SD	4.2149
SE	0.5961

39	0
40	0
41	0
42	0
43	0
44	1
45	0
46	5
47	2
48	5
49	0
50	0
Average	**2.9**
Count	50
SD	5.504
SE	0.778

Supplementary Table S5. Presence of virulence-associated genes (denoted with a dot), characteristics of LPS and phylogenetic group of the studied *E. coli* strains.

	Fecal strains										Uropathogenic strains										Control strains			
**Virulence-associated gene**	BJ16	BJ23	BJ30	BJ45	BJ50	BJ51	BJ65	BJ69	BJ95	BJ97	DL1	DL18	DL31	DL53	DL75	DL80	DL87	DL95	DL102	HS16	SE15	MG1655	J96	536
cnf1			●								●	●	●	●					●				●	
hlyA			●								●	●	●	●					●	●			●	●
usp		●	●			●	●	●	●	●	●	●	●	●	●			●	●	●	●		●	●
clbA		●	●					●		●	●	●	●	●					●	●			●	●
clbQ		●	●					●		●	●	●	●	●					●	●			●	●
vat		●	●					●	●	●	●	●	●	●				●	●	●			●	●
fimH		●	●	●	●	●	●	●	●	●	●	●	●	●	●	●	●	●	●	●	●	●	●	●
papGII						●			●			●			●			●						
papGIII			●								●		●	●					●				●	●
afa/draBC																								
sfaDE		●	●					●		●	●	●	●	●					●				●	●
iha								●				●		●						●				
yfcV		●	●			●		●	●	●	●	●	●	●	●			●	●	●	●		●	●
fyuA		●	●			●		●	●	●	●	●	●	●	●			●	●	●	●		●	●
hbp		●								●														
ireA		●				●			●			●			●			●						
picU													●						●	●				
iucD		●				●		●	●	●		●			●			●		●				
iroN		●	●			●		●	●	●	●	●	●	●	●			●	●	●			●	●
kpsMTII		●	●			●		●	●	●	●	●		●	●			●	●	●	●			
ompT		●	●			●		●	●	●	●	●	●	●	●		●	●	●	●	●	●	●	●
ompT-APEC		●				●		●	●	●					●			●						
tcpC			●					●			●	●	●	●					●				●	●
traT		●		●		●			●	●		●			●			●		●	●		●	
iss		●				●			●						●			●						
neuB		●	●			●			●	●				●	●			●						
ibeA		●								●									●					
**Core LPS**	K-12	R1	R1	R1	K12	R1	K12	R1	R1	R1	R1	R1	R1	R1	R1	R1	K12	R1	K12	R1	K12	K-12	R1	R1
**O - serotype**		O-18				O-1		O-75	O-1	O-18	O-6	O4	O-6		O-2			O-1	O75	O25	O-16	O-16	O-4	O6
**LPS - type**	smooth	smooth	smooth	smooth	smooth	smooth	smooth	smooth	smooth	smooth	smooth	smooth	rough	smooth	smooth	smooth	rough	smooth	rough	smooth	smooth	rough	smooth	smooth
**Phylogeny triplex PCR**	A	B2	B2	A	A	B2	A	B2	B2	B2	B2	B2	B2	B2	B2	A	A	B2	B2	A	B2	A	B2	B2
**Phylogeny quadruplex PCR**	A	B2	B2	A	A	B2	A	B2	B2	B2	B2	B2	B2	B2	B2	A	A	B2	B2	B2	B2	A	B2	B2

● gene present

Supplementary Table S6A. P (two-tail) values obtained with automated Fischer's exact test computer software (presented with four decimals).

	**Commensal group I**	**Low pathogenic group II**	**Highly pathogenic group III**
cnf	0.0686	1.0000	0.0086
hlyA	0.0894	0.6146	0.0037
usp	1.0000	0.0785	0.1300
clbAQ	0.2203	0.3168	0.0046
papGII	0.3168	0.5440	1.0000
papGIII	0.0686	1.0000	0.0086
afa/draBC	1.0000	1.0000	1.0000
sfaDE	0.0995	0.3271	0.0010
fyuA	1.0000	0.0145	0.0648
hbp	1.0000	1.0000	0.5072
iha	1.0000	0.5440	0.5520
ireA	0.6404	0.2801	1.0000
picU	1.0000	1.0000	1.0000
iucD	0.0894	0.1181	0.6687
iroN	1.0000	0.0277	0.0538
kpsMTII	0.6802	0.1222	0.6529
ompT	0.3168	0.0023	0.2721
ompT-APEC	0.0686	0.2721	0.6245
tcpC	0.0894	0.6146	0.0037
traT	0.4136	0.3271	1.0000
iss	0.3168	0.5440	1.0000
neuB	0.6668	0.1304	0.6466
ibeA	1.0000	1.0000	0.1937
fimH	1.0000	0.2083	1.0000
yfc	1.0000	0.0145	0.0648
vat	0.6802	0.1222	0.0188
**R1**	1.0000	0.1265	0.6245
**R2**	1.0000	1.0000	1.0000
**R3**	1.0000	1.0000	1.0000
**R4**	1.0000	1.0000	1.0000
**K-12**	1.0000	0.1265	0.6245
**O1**	0.2174	1.0000	0.5296
**O2**	1.0000	1.0000	1.0000

**O4**	0.4783	1.0000	0.0761
**O6**	1.0000	0.5212	1.0000
**O7**	1.0000	1.0000	1.0000
**O12**	1.0000	1.0000	1.0000
**O15**	1.0000	1.0000	1.0000
**O16**	0.4783	1.0000	1.0000
**O18**	1.0000	1.0000	0.5072
**O25**	1.0000	1.0000	1.0000
**O75**	1.0000	1.0000	0.5072
**smooth**	0.5901	0.5440	1.0000
**semi-rough**	1.0000	1.0000	1.0000
**rough**	0.5901	0.5440	1.0000
**Phylogroup A (triplex)**	1.0000	0.0277	0.0538
**Phylogroup B2 (triplex)**	1.0000	0.0277	0.0538
**Phylogroup A (quadruplex)**	1.0000	0.0145	0.0648
**Phylogroup B2 (quadruplex)**	1.0000	0.0145	0.0648

Supplementary Table S6B. Data obtained after Bonferroni correction applied to the data in the Supplementary Table S6A (presented with four decimals).

	**Commensal group I**	**Low pathogenic group II**	**Highly pathogenic group III**
cnf	0.2059	3.0000	0.0258
hlyA	0.2681	1.8439	0.0112
usp	3.0000	0.2355	0.3901
clbAQ	0.6610	0.9503	0.0137
papGII	0.9503	1.6321	3.0000
papGIII	0.2059	3.0000	0.0258
afa/draBC	3.0000	3.0000	3.0000
sfaDE	0.2986	0.9814	0.0029
fyuA	3.0000	0.0435	0.1944
hbp	3.0000	3.0000	1.5217
iha	3.0000	1.6321	1.6561
ireA	1.9212	0.8402	3.0000
picU	3.0000	3.0000	3.0000
iucD	0.2681	0.3542	2.0061
iroN	3.0000	0.0830	0.1614
kpsMTII	2.0405	0.3666	1.9588
ompT	0.9503	0.0068	0.8162
ompT-APEC	0.2059	0.8162	1.8736
tcpC	0.2681	1.8439	0.0112
traT	1.2409	0.9814	3.0000
iss	0.9503	1.6321	3.0000
neuB	2.0005	0.3913	1.9399
ibeA	3.0000	3.0000	0.5810
fimH	3.0000	0.6250	3.0000
yfc	3.0000	0.0435	0.1944
vat	2.0405	0.3666	0.0563
**R1**	3.0000	0.3794	1.8736
**R2**	3.0000	3.0000	3.0000
**R3**	3.0000	3.0000	3.0000
**R4**	3.0000	3.0000	3.0000
**K-12**	3.0000	0.3794	1.8736
**O1**	0.6522	3.0000	1.5889
**O2**	3.0000	3.0000	3.0000

**O4**	1.4348	3.0000	0.2283
**O6**	3.0000	1.5637	3.0000
**O7**	3.0000	3.0000	3.0000
**O12**	3.0000	3.0000	3.0000
**O15**	3.0000	3.0000	3.0000
**O16**	1.4348	3.0000	3.0000
**O18**	3.0000	3.0000	1.5217
**O25**	3.0000	3.0000	3.0000
**O75**	3.0000	3.0000	1.5217
**smooth**	1.7702	1.6321	3.0000
**semi-rough**	3.0000	3.0000	3.0000
**rough**	1.7702	1.6321	3.0000
**Phylogroup A (triplex)**	3.0000	0.0830	0.1614
**Phylogroup B2 (triplex)**	3.0000	0.0830	0.1614
**Phylogroup A (quadruplex)**	3.0000	0.0435	0.1944
**Phylogroup B2 (quadruplex)**	3.0000	0.0435	0.1944
